# Detection of oseltamivir‐resistant zoonotic and animal influenza A viruses using the rapid influenza antiviral resistance test

**DOI:** 10.1111/irv.12661

**Published:** 2019-06-11

**Authors:** Erin N. Hodges, Vasiliy P. Mishin, Juan De la Cruz, Zhu Guo, Ha T. Nguyen, Eric Fallows, James Stevens, David E. Wentworth, Charles Todd Davis, Larisa V. Gubareva

**Affiliations:** ^1^ Influenza Division Centers for Disease Control and Prevention (CDC) Atlanta Georgia; ^2^ CNI Advantage Atlanta Georgia; ^3^ Battelle Memorial Institute Atlanta Georgia; ^4^ Becton, Dickinson and Company Research Triangle Park North Carolina

**Keywords:** antiviral drugs, avian, influenza A virus, resistance, zoonotic

## Abstract

Mutations in the influenza virus neuraminidase (NA) that cause reduced susceptibility to the NA inhibitor (NAI) oseltamivir may occur naturally or following antiviral treatment. Currently, detection uses either a traditional NA inhibition assay or gene sequencing to identify known markers associated with reduced inhibition by oseltamivir. Both methods are laborious and require trained personnel. The influenza antiviral resistance test (iART), a prototype system developed by Becton, Dickinson and Company for research use only, offers a rapid and simple method to identify such viruses. This study investigated application of iART to influenza A viruses isolated from non‐human hosts with a variety of NA subtypes (N1‐N9).

## 1 INTRODUCTION

Zoonotic and animal influenza A viruses pose a significant threat to public health; they can cause severe disease in humans with little protection afforded by seasonal vaccination due to antigenic differences.^1^ NAIs are routinely used to treat individuals infected with influenza viruses, regardless of subtype, and the oseltamivir is the most commonly prescribed anti‐influenza therapeutic. Antiviral resistance can emerge in nature or following treatment with NAIs through changes to the surface antigen NA that affect neuraminidase inhibitor (NAI) binding. Such changes may cause resistance to one or more NAIs.^2^

While NA gene sequence analysis is often used to screen viruses for established markers of resistance, genetic analysis cannot identify viruses carrying new molecular markers, or assess the degree of reduced susceptibility. Thus, phenotypic NAI assays are commonly used to assess viral susceptibility to NAIs.^3^ In these assays, virus is diluted to a targeted level of NA activity and tested against serially diluted NAI to determine an IC_50_, the drug concentration needed to inhibit 50% of NA activity. To report the results for seasonal influenza A viruses, the fold change of the test virus is calculated by comparison to a reference IC_50_ value, either a subtype‐specific median or the IC_50_ of a control virus lacking the NA change.^4^ However, this approach cannot be readily applied to testing and reporting of non‐seasonal influenza virus susceptibility to NAIs because of difficulty of acquiring and testing large numbers of each distinct subtype and wide range of genetic lineages within each subtype. Moreover, NAI results require careful interpretation, as laboratory correlates of clinically relevant resistance have not been established, except for viruses carrying an N1 NA with the H275Y substitution.^5^ Infections caused by viruses displaying reduced inhibition (RI) or highly reduced inhibition (HRI) phenotypes may be more difficult to control by therapeutic intervention, which can lead to prolonged illness and virus shedding.^6^

Simple and rapid assays that can be used by surveillance laboratories and in clinical settings are needed to detect viruses with reduced susceptibility to NAIs. As previously reported, the prototype influenza antiviral resistance test (iART), developed by BD Technologies (BARDA Contract HHSO100201300008C), is able to phenotypically detect seasonal influenza viruses that display RI/HRI by oseltamivir.^7^ This assay compares influenza‐specific sialidase (NA) activity with and without a single drug concentration, requires only 1 hour, and does not need extensive training to carry out. Here, we present similar findings for zoonotic and animal influenza viruses.

## 2 COMPARISON OF iART TO NAI ASSAY

To verify the ability of iART to efficiently detect NA enzymatic activity and inhibition by oseltamivir of various subtypes (N1 through N9), a variety of zoonotic and animal influenza viruses were tested. This included viruses (n = 45) isolated from wild birds, poultry, a domestic cat, and zoonotic human infections propagated in MDCK cells or fertilized chicken eggs (Table 1). NA sequence analysis did not identify known or suspected markers of resistance to oseltamivir (Table S1). Viruses were tested using both the fluorescence‐based NAI and iART assays, as previously described.^4^ All virus isolates were found to be susceptible to inhibition by oseltamivir in the iART assay (*R*‐factor ≤0.70). In the NAI assay, all calculated IC_50_ values were in the nanomolar/subnanomolar range; some differences among subtypes were observed, as expected, with the greatest IC_50_ value observed for N8 viruses and the lowest for N2 viruses (Table 1). The median IC_50_ for all subtypes (calculated using an average IC_50_ for each subtype) was determined to be 0.48 nmol/L (Table S2). Using the median IC_50_, the fold change was calculated for each isolate. As expected, all tested viruses were determined to be normally inhibited (NI) by oseltamivir, and, therefore, susceptible to this drug, according to the criteria implemented by the Expert Working Group on Antiviral Susceptibility for the WHO Global Influenza Surveillance and Response System^5^ (<10‐fold increase compared to the median IC_50_). The data from the gold standard NAI assay showed good correlation with the results obtained using iART, verifying the test's ability to detect NA enzymatic activity and inhibition by oseltamivir for non‐seasonal influenza viruses.

**Table 1:** Zoonotic and avian influenza A viruses of the N1‐N9 neuraminidase (NA) subtypes and NA inhibitor (NAI) activity

Virus name	HA subtype	NA subtype	NAI assay^a^		iART *R*‐factor^c^
			IC_50_ (nmol/L)	Fold change^b^	
A/Iowa/33/2017	H1v	N1	0.12	0.25	0.08
A/Ohio/09/2015	H1v	N1	0.39	0.80	0.06
A/Vietnam/1203/2004	H5	N1	0.76	0.58	0.11
A/Alberta/01/2014	H5	N1	1.24	0.96	0.03
A/duck/Vietnam/NCVD‐680/2011	H5	N1	1.51	1.17	0.08
A/guinea fowl/Italy/407/2008	H7	N1	2.94	2.26	0.07
A/Michigan/09/2007	H3v	N2	0.27	0.21	0.22
A/Ohio/83/2012	H3v	N2	0.41	0.31	0.22
A/Iowa/04/2013	H3v	N2	0.54	0.42	0.25
A/Ohio/02/2014	H3v	N2	0.49	0.38	0.31
A/Ohio/4319/2014	H3v	N2	0.54	0.42	0.19
A/Wisconsin/24/2014	H3v	N2	0.57	0.44	0.24
A/Michigan/83/2016	H3v	N2	0.32	0.25	0.23
A/Michigan/84/2016	H3v	N2	0.30	0.23	0.31
A/Ohio/27/2016	H3v	N2	0.24	0.19	0.23
A/Ohio/28/2016	H3v	N2	0.27	0.21	0.17
A/northern pintail/Washington/40964/2014	H5	N2	0.30	0.23	0.03
A/New York/108/2016	H7	N2	0.32	0.25	0.14
A/feline/New York/16‐040082‐1/2016	H7	N2	1.11	0.85	0.41
A/chicken/Bangladesh/OP‐4/2013	H9	N2	0.38	0.29	0.11
A/chicken/Bangladesh/3C‐44/2014	H9	N2	0.52	0.40	0.02
A/chicken/Vietnam/NCVD‐LS52/2016	H9	N2	2.65	2.04	0.02
A/duck/Bangladesh/19D691/2016	H11	N2	0.67	0.51	0.12
A/chicken/Mexico/8201/12	H7	N3	0.78	0.60	0.08
A/duck/Bangladesh/18D659/2016	H1	N4	1.66	1.28	0.14
A/nomadic duck/Bangladesh/740/2011	H2	N4	3.03	2.33	0.18
A/duck/Bangladesh/17D747/2016	H3	N5	2.16	1.67	0.07
A/duck/Peru/MM17/08	H4	N5	2.35	1.81	0.2
A/goose/Bangladesh/19D820/2017	H5	N6	0.78	0.60	0.37
A/duck/Bangladesh/19D849/2017	H5	N6	1.01	0.78	0.37
A/duck/Bangladesh/19D857/2017	H5	N6	1.01	0.78	0.26
A/chicken/Vietnam/NCVD‐16A26/2016	H5	N6	3.65	2.81	0.09
A/duck/Vietnam/NCVD‐90911/2013	H6	N6	1.57	1.21	0.1
A/waterfowl/Bangladesh/12301/2013	H6	N7	0.76	0.58	0.36
A/duck/Bangladesh/18D769/2017	H6	N7	1.10	0.85	0.16
A/duck/Bangladesh/20D677/2016	H3	N8	6.83	5.26	0.12
A/duck/Vietnam/NCVD‐ND4V3P/2016	H3	N8	2.41	1.85	0.08
A/gyrfalcon/Washington/41088‐6/2014	H5	N8	1.68	1.29	0.09
A/turkey/Indiana/1403/2016	H7	N8	3.95	3.04	0.14
A/Jiangxi/09037/2014	H10	N8	2.56	1.97	0.35
A/Shanghai/1/2013	H7	N9	0.78	0.60	0.36
A/Taiwan/1/2013	H7	N9	0.95	0.73	0.08
A/Hong Kong/4553/2016	H7	N9	1.43	1.10	0.08
A/Hong Kong/61/2016	H7	N9	1.14	0.88	0.08
A/Hong Kong/125/2017	H7	N9	1.22	0.94	0.26
Overall range	N1‐N9		0.09‐2.53	0.19‐5.26	0.02‐0.41

a Tested using the US Centers for Disease Control and Prevention standardized fluorescence‐based NAI assay.

b Fold change shows the fold increase in IC_50_ value of the test virus compared with the median IC_50_ for all subtypes.

c *R*‐factor: ratio of chemiluminescent signal intensity generated by viral NA activity on the substrate with and without inhibitor (ie, oseltamivir carboxylate).

To verify that iART was able to detect reduced susceptibility to oseltamivir of avian and zoonotic viruses, nine virus isolates with NA amino acid substitutions known to affect oseltamivir susceptibility were tested by both the NAI and iART assays (Table 2). Calculated IC_50_ values were compared to control viruses that lacked the NA substitution, as well as to the median IC_50_ value calculated above. The median IC_50_ fold change calculation is necessary when a matching wild‐type virus is not available or a virus with an unknown NA sequence is tested. The method of fold change did not change the interpretation for eight of nine viruses (Table 2). One isolate (Table 2, A/Vietnam/HN30408/2005 clone 1) was interpreted as having RI using the fold change determined with the control virus IC_50_, normal inhibition (NI) using the fold change determined with the median IC_50_, and an *R*‐factor that was below the pre‐set threshold of 0.70 (0.57). Two viruses (Table 2, A/Ohio/88/2012 and A/Taiwan/1/2013 clone 3) tested as RI by NAI with an *R*‐factor in iART near the threshold (0.62, 0.66). The other six viruses that had RI or HRI phenotypes by the NAI assay showed *R*‐factors above the ≥0.70 threshold in the iART assay.

**Table 2:** Zoonotic and avian influenza A viruses with neuraminidase (NA) substitutions conferring (highly) reduced inhibition by oseltamivir

Virus name	Subtype	NA amino acid substitution^a^		NAI assay^b^				iART	
		Straight N9 numbering	N2 numbering	IC_50_ (nmol/L) Mean ± SD	Fold change vs control virus	Fold change vs median of all subtypes	Interpretation^c^	*R*‐factor^d^	Result
A/Vietnam/HN30408/2005 clone 1	H5N1 Clade 1	n/a	N294S	2.76 ± 0.37	10	6	RI/NI	0.57	Non‐resistant
A/Vietnam/HN30408/2005 clone 2	H5N1 Clade 1	n/a	H274Y	189.94 ± 36.95	687	396	HRI	4.06	Resistant
A/duck/Vietnam/NCVD‐664/2010	H5N1 Clade 2.3.2.1	n/a	H274Y	259.91 ± 46.79	466	541	HRI	6.37	Resistant
A/Ohio/88/2012	H3N2v	n/a	S247P	5.22 ± 0.97	54	11	RI	0.62	Non‐resistant
A/Taiwan/1/2013 clone 1	H7N9	E115V	E119V	27.79 ± 2.86	79	58	RI	2.10	Resistant
A/Taiwan/1/2013 clone 2	H7N9	I219R	I222R	14.97 ± 6.73	43	31	RI	1.00	Resistant
A/Taiwan/1/2013 clone 3	H7N9	I219K	I222K	8.82 ± 0.27	24	18	RI	0.66	Non‐resistant
A/Taiwan/1/2013 clone 4	H7N9	R289K	R292K	>1000	>3000	>2000	HRI	9.84	Resistant
A/Shanghai/1/2013 clone 1	H7N9	R289K	R292K	>1000	>3000	>2000	HRI	8.39	Resistant

a NA amino acid substitution position shown using both straight numbering and N2 subtype numbering.

b Tested using the US Centers for Disease Control and Prevention standardized fluorescence‐based NAI assay. Mean and standard deviation (SD) of at least three independent experiments shown; fold change shows the fold increase in IC_50_ value of the test virus compared with a control virus IC_50_ value (for the virus lacking the amino acid substitution) and using the median IC_50_ of all subtypes.

c Criteria for interpreting NAI assay results based on IC_50_ fold increase compared with the control virus/median IC_50_ value: normal inhibition (NI) <10‐fold, reduced inhibition (RI) 10‐ to 100‐fold, and highly reduced inhibition (HRI) >100‐fold.

d *R*‐factor: ratio of chemiluminescent signal intensity generated by viral NA activity with and without inhibitor (ie, oseltamivir carboxylate). *R*‐factor interpretation based on pre‐set cutoff for influenza A (resistance is ≥0.70).

A wide range of *R*‐factors were observed, which correlated with the range of fold differences determined by NAI assay (Figure S1). Viruses with the highest *R*‐factors (ie, >4.0) were also identified as having HRI by the NAI assay. Viruses with RI or fold change values near the 10‐fold cutoff had *R*‐factors near the 0.70 threshold. These results demonstrated that any virus reported as resistant by iART would have RI/HRI by NAI. Non‐resistant viruses, particularly those with elevated *R*‐factors, also showed some reduced inhibition by oseltamivir. With further testing and refinement of the *R*‐factor threshold, iART may be able to differentiate between RI and HRI viruses in the future. Alternatively, any specimen with an *R*‐factor above 0.50 could be flagged for sequence analysis and additional testing in the NAI assay. None of the wild‐type type viruses shown in Table 1 or seasonal viruses reported previously would be flagged as having potentially reduced susceptibility using a lower threshold for type A viruses.^7^

## 3 RECOMBINANT N9 PROTEINS WITH KNOWN MARKERS OF RI/HRI BY OSELTAMIVIR

Amino acid substitutions known to reduce susceptibility to oseltamivir E119V, I222K/R, H274Y, R292K, and R371K (N2 numbering) have been detected in the NA of A(H7N9) viruses isolated from humans.^8^ In addition, I222T was detected in an A(H7N9) virus isolated from a non‐human primate after oseltamivir treatment.^9^ To determine whether iART is able to identify NA with these changes as resistant to oseltamivir, the respective recombinant N9 (rN9) proteins were generated using the A/Shanghai/2/2013 NA as a backbone, as previously described.^10^ The use of recombinant protein allows testing of amino acid changes that reduce enzymatic activity in addition to reducing susceptibility to NAIs, including R292K (R289K in N9 straight numbering), the most commonly identified NA change detected in H7N9 human cases. The *R*‐factors of rN9 proteins carrying substitutions E119V, I222K/R, H274Y, R292K, or R371K categorized them as resistant to oseltamivir and correlated with NAI assay outcomes (Table 3). The range of *R*‐factors also correlated with the range of IC_50 _values (Figure S1); all rN9 with *R*‐factors above 2.0 were identified as having HRI by the NAI assay. The rN9 protein with I222T was identified as non‐resistant by iART. In the NAI assay, the fold change conferred by this substitution was below the threshold of 10, further confirming the correlation between the two assays.

**Table 3:** Recombinant neuraminidase (NA) proteins of A/Shanghai/2/2013 (H7N9) with substitutions conferring (highly) reduced inhibition by oseltamivir

NA amino acid substitution^a^		NAI assay^b^			iART	
Recombinant N9 (straight N9 numbering)	N2 numbering	IC_50 _(nmol/L) Mean ± SD	Fold change	Interpretation^c^	*R*‐factor^d^	Result
Shanghai/2/2013	None	0.31 ± 0.02	1	NI	0.09 ± 0.08	Non‐resistant
E115V	E119V	55.18 ± 1.02	176	HRI	2.10 ± 0.22	Resistant
I219K	I222K	14.89 ± 0.39	48	RI	0.88 ± 0.14	Resistant
I219R	I222R	27.01 ± 0.62	86	RI	1.34 ± 0.05	Resistant
I219T	I222T	2.79 ± 0.04	9	NI	0.23 ± 0.05	Non‐resistant
H271Y	H274Y	36.71 ± 0.86	117	HRI	2.04 ± 0.10	Resistant
R289K	R292K	>1000	>3192	HRI	9.48 ± 0.14	Resistant
R367K	R371K	24.56 ± 1.26	78	RI	1.71 ± 0.20	Resistant

a NA amino acid substitution position is shown using both straight numbering and N2 subtype numbering.

b Tested using the US Centers for Disease Control and Prevention standardized fluorescence‐based NAI assay. Mean and standard deviation (SD) of at least three independent experiments shown; fold change shows the fold increase in IC_50_ value of the test recombinant NA protein compared with the A/Shanghai/2/2013 NA protein IC_50_ value.

c Criteria for interpreting NAI assay results based on the fold increase in IC_50_ value of the test NA compared with the wild‐type A/Shanghai/2/2013 NA protein IC_50_ value: normal inhibition (NI) <10‐fold, reduced inhibition (RI) 10‐ to 100‐fold, and highly reduced inhibition (HRI) >100‐fold.

d *R*‐factor: ratio of chemiluminescent signal intensity generated by viral NA activity on the substrate with and without inhibitor (ie, oseltamivir carboxylate). Mean and standard deviation of *R*‐factors from three independent experiments. *R*‐factor interpretation based on pre‐set cutoff for influenza A (resistance is ≥0.70).

## 4 IART VS NAI ASSAY UNDER LOW PH CONDITIONS (PH 5.3 VS 6.8)

As mentioned above, R292K is the most commonly reported NA marker in oseltamivir‐treated patients infected with A(H7N9) viruses. In addition, this change is also known to reduce enzymatic activity, making detection of drug resistance difficult using the standard NAI assay due to insufficient activity for testing or wild‐type activity masking resistance.^11^ It was previously reported that detection of R292K viruses could be improved by NAI testing at an acidic pH.^12^ To confirm this finding, testing was performed on a highly pathogenic avian influenza A(H7N9) isolate, A/Taiwan/1/2017, containing the R292K substitution. At a standard pH of 6.8, the NAI assay was unable to test this virus isolate as NA activity was below the threshold needed for testing (Table 4). At a pH of 5.3, however, this virus had sufficient NA activity and displayed an HRI phenotype. Notably, iART was able to detect resistance caused by R292K, without modifying the pH conditions of the assay. We previously showed that clinical specimens can be tested directly by iART, even when NA activity is insufficient for testing by NAI.^7^ These results confirm and extend those findings and suggest the greater sensitivity of iART to detect resistance in low‐activity NA viruses.

**Table 4:** Outcome of influenza antiviral resistance test (iART) vs NAI assay testing under low pH (pH 5.3)

Virus name	Subtype	NAI assay				iART	
		Standard pH 6.8		Modified pH 5.3			
		IC_50_ (nmol/L)	IC_50_ (nmol/L)	Fold	Result^b^	*R*‐factor^c^	Result
A/Taiwan/01/2017	HPAI H7N9	N/A^a^	>1000	>1500	HRI	9.90 ± 1.43	Resistant

a N/A: Not available because NA enzyme activity level was insufficient for testing.

b Criteria for reporting NAI assay results based on the fold increase in IC_50_ value of the test virus compared with the IC_50_ value of a control virus without the R292K substitution: normal inhibition (NI) <10‐fold, reduced inhibition (RI) 10‐ to 100‐fold, and highly reduced inhibition (HRI) >100‐fold.

c *R*‐factor: ratio of chemiluminescent signal intensity generated by viral NA activity on the substrate with and without inhibitor (ie, oseltamivir carboxylate). Mean and standard deviation of *R*‐factors from three independent experiments. *R*‐factor interpretation based on pre‐set cutoff for influenza A (resistance is ≥0.70).

Influenza antiviral resistance test is a rapid and sensitive phenotypic assay for the detection of influenza viruses with reduced inhibition by oseltamivir. Unlike sequence‐based methods, iART provides phenotypic data that are valuable for the identification of viruses carrying both known and unknown molecular markers associated with reduced susceptibility. As new animal and zoonotic subtype viruses emerge, it is critical to determine their drug phenotype rapidly so that public health authorities and clinicians can better assess treatment options. iART is currently not commercially available, though another influenza‐specific assay (QFlu Combo Test by Cellex) uses a similar principal of oseltamivir resistance detection. The future availability of iART depends on demand for point of care assays to detect antiviral resistance.

While the gold standard NAI assay continues to be the assay of choice for surveillance laboratories, it is cumbersome and requires highly trained personnel. iART provides an alternative, simple method for detecting oseltamivir‐resistant viruses using a small and portable device with built‐in software for data interpretation. Viruses detected by iART with elevated *R*‐factors can be flagged for genetic analysis and comprehensive phenotypic evaluation. This design and ease of use may allow oseltamivir susceptibility testing in locations currently unable to carry out the NAI assay.
