# Supplementary material for: Detection of oseltamivir‐resistant zoonotic and animal influenza A viruses using the rapid influenza antiviral resistance test
Source: Influenza Other Respir Viruses. 2019 Jun 11;13(5):522–7. doi: 10.1111/irv.12661 (PMC6692545; doi:10.1111/irv.12661)
Supplement: Supplementary file 2 [file IRV-13-522-s002.docx]

**Supplemental Table 1.** GISAID accession numbers of viral genome sequences analyzed in this study.

| **Virus Name** | **HA** | **NA** | **GISAID ID** |
| --- | --- | --- | --- |
| A/Iowa/33/2017 | H1v | N1 | EPI_ISL_329966 |
| A/Ohio/09/2015 | H1v | N1 | EPI_ISL_179403 |
| A/Vietnam/1203/2004 | H5 | N1 | EPI_ISL_21080 |
| A/Alberta/01/2014 | H5 | N1 | EPI_ISL_154130 |
| A/duck/Vietnam/NCVD-680/2011 | H5 | N1 | EPI_ISL_136101 |
| A/Vietnam/HN30408/2005 | H5 | N1 | EPI_ISL_138227 |
| A/duck/Vietnam/NCVD-664/2010 | H5 | N1 | EPI_ISL_136100 |
| A/guineafowl/Italy/407/2008 | H7 | N1 | EPI_ISL_60058 |
| A/Ohio/83/2012 | H3v | N2 | EPI_ISL_129651 |
| A/Iowa/04/2013 | H3v | N2 | EPI_ISL_159598 |
| A/Ohio/02/2014 | H3v | N2 | EPI_ISL_165317 |
| A/Ohio/4319/2014 | H3v | N2 | EPI_ISL_166393 |
| A/Wisconsin/24/2014 | H3v | N2 | EPI_ISL_170376 |
| A/Michigan/83/2016 | H3v | N2 | EPI_ISL_232048 |
| A/Michigan/84/2016 | H3v | N2 | EPI_ISL_232047 |
| A/Ohio/27/2016 | H3v | N2 | EPI_ISL_232044 |
| A/Ohio/28/2016 | H3v | N2 | EPI_ISL_232045 |
| A/northern pintail/Washington/40964/2014 | H5 | N2 | EPI_ISL_238031 |
| A/New York/108/2016 | H7 | N2 | EPI_ISL_253575 |
| A/feline/New York/16-040082-1/2016 | H7 | N2 | EPI_ISL_260817 |
| A/chicken/Bangladesh/OP-4/2013 | H9 | N2 | EPI_ISL_218649 |
| A/chicken/Bangladesh/3C-44/2014 | H9 | N2 | EPI_ISL_333970 |
| A/chicken/Vietnam/NCVD-LS52/2016 | H9 | N2 | EPI_ISL_333973 |
| A/duck/Bangladesh/19D691/2016 | H11 | N2 | EPI_ISL_333094 |
| A/chicken/Mexico/8201/12 | H7 | N3 | EPI_ISL_333968 |
| A/duck/Bangladesh/18D659/2016 | H1 | N4 | EPI_ISL_333097 |
| A/nomadic duck/Bangladesh/740/2011 | H2 | N4 | EPI_ISL_149696 |
| A/duck/Bangladesh/17D747/2016 | H3 | N5 | EPI_ISL_333102 |
| A/duck/Peru/MM17/2007 | H4 | N5 | EPI_ISL_189977 |
| A/goose/Bangladesh/19D820/2017 | H5 | N6 | EPI_ISL_333132 |
| A/duck/Bangladesh/19D849/2017 | H5 | N6 | EPI_ISL_332948 |
| A/duck/Bangladesh/19D857/2017 | H5 | N6 | EPI_ISL_332804 |
| A/chicken/Vietnam/NCVD-16A26/2016 | H5 | N6 | EPI_ISL_333975 |
| A/duck/Vietnam/NCVD-90911/2013 | H6 | N6 | EPI_ISL_333969 |
| A/waterfowl/Bangladesh/12301/2013 | H6 | N7 | EPI_ISL_165810 |
| A/duck/Bangladesh/18D769/2017 | H6 | N7 | EPI_ISL_333138 |
| A/duck/Bangladesh/20D677/2016 | H3 | N8 | EPI_ISL_333104 |
| A/duck/Vietnam/NCVD-ND4V3P/2016 | H3 | N8 | EPI_ISL_333974 |
| A/gyrfalcon/Washington/41088-6/2014 | H5 | N8 | EPI_ISL_173878 |
| A/turkey/Indiana/1403/2016 | H7 | N8 | EPI_ISL_218653 |
| A/Jiangxi/09037/2014 | H10 | N8 | EPI_ISL_162062 |
| A/Shanghai/1/2013 | H7 | N9 | EPI_ISL_138737 |
| A/Taiwan/1/2013 | H7 | N9 | EPI_ISL_140356 |
| A/Hong Kong/4553/2016 | H7 | N9 | EPI_ISL_333971 |
| A/Hong Kong/61/2016 | H7 | N9 | EPI_ISL_333972 |
| A/Hong Kong/125/2017 | H7 | N9 | EPI_ISL_259269 |
| A/Taiwan/01/2017 | HP-H7 | N9 | EPI_ISL_248778 |

**Supplementary Table 2. Average NAI IC50 by subtype.**

| **NA Subtype** | **# Tested** | **Average** |
| --- | --- | --- |
| N1 | 6 | 0.48 |
| N2 | 17 | 0.22 |
| N3 | 1 | 0.29 |
| N4 | 2 | 0.87 |
| N5 | 2 | 0.84 |
| N6 | 5 | 0.59 |
| N7 | 2 | 0.34 |
| N8 | 5 | 1.29 |
| N9 | 5 | 0.41 |
| TOTAL | 45 |  |
| Median Subtype IC50 | | 0.48 |
